# Alterations in brain myelination at early-stage schizophrenia detected by macromolecular proton fraction MRI

**DOI:** 10.1192/j.eurpsy.2023.343

**Published:** 2023-07-19

**Authors:** E. Krupina, A. Manzhurtsev, M. Ublinskiy, O. Bozhko, G. Mamedova, V. Ushakov, N. Zakharova, V. Yarnykh, D. Andreyuk, M. Shlyapnikov, G. Kostyuk, T. Akhadov

**Affiliations:** 1 National Research Nuclear University MEPhI; 2 Clinical and Research Institute of Emergency Pediatric Surgery and Trauma; 3 Emanuel Institute of Biochemical Physics of the Russian Academy of Sciences; 4 Moscow State University; 5 Psychiatric Clinical Hospital 1 named N.A. Alekseev.; 6 Institute for Advanced Brain Studies, Moscow State University, Moscow, Russian Federation; 7 Radiology at the University of Washington, Seattle, United States

## Abstract

**Introduction:**

There is evidence that cerebral myelination is impaired in schizophrenia. The purpose of this study is to find the myelin content changes in the brain structures of patients with early-stage schizophrenia using the macromolecular proton fraction (MPF) method, and also to evaluate the differences in the myelination of these structures.

**Objectives:**

To measure MPF in the brain structures of schizophrenia patients

**Methods:**

Forty-five subjects, 22 controls (10m+12f, 31.6±9.7 y.o.) and 23 schizophrenia patients (F20.0, 11m+12f, 31.5±5.1 y.o.). Philips Achieva dStream 3T MRI scanner, standard head coil. The magnetization transfer (TR=20 ms, TE=4.60 ms, FA=10°), T1-weighted (TR=20 ms, TE=4.60 ms, FA=20°) and PD-weighted (TR=20 ms, TE=4.60 ms, FA=4°) were acquired. The MPF maps were reconstructed using home-made software. In FSL, non-brain structures were removed and MPF maps were registered to a standard MNI152 1 mm atlas. Harvard Oxford Cortical and Subcortical atlases were used to select areas of interest. T-test was used in search for between-group differences.

**Results:**

A 3% decrease in myelination in schizophrenia was observed in whole cerebral cortex p = 0.03) and cerebral white matter (p=0.02). Trends to cortical demyelination were found: paracingular cortex (p=0.06), anterior (p=0.1) and posterior cingulate cortex (p=0.07). No myelination disorders were detected in the cerebellum.

**Image:**

**Figure:**
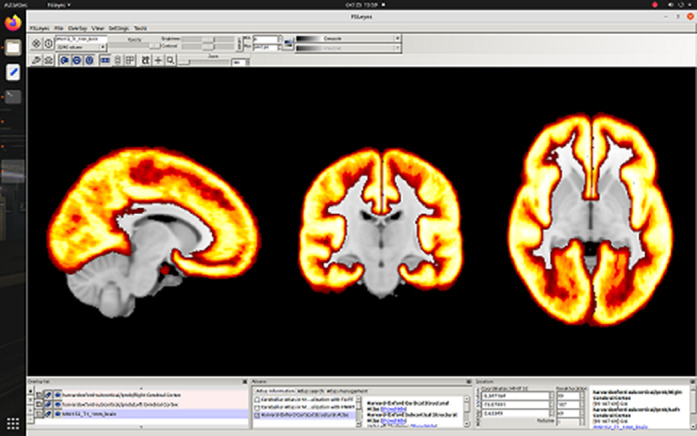

**Image 2:**

**Figure:**
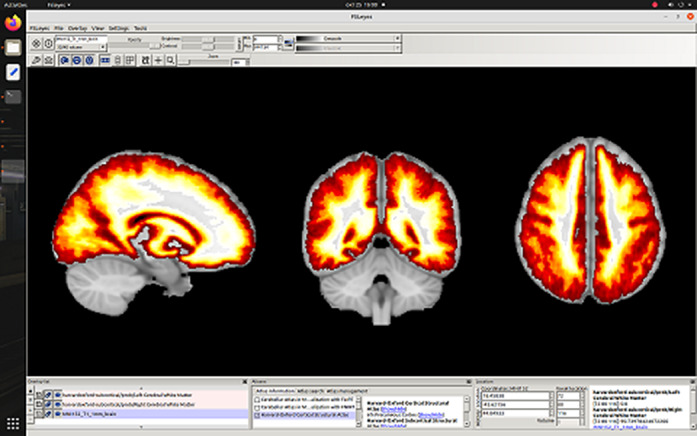

**Image 3:**

**Figure:**
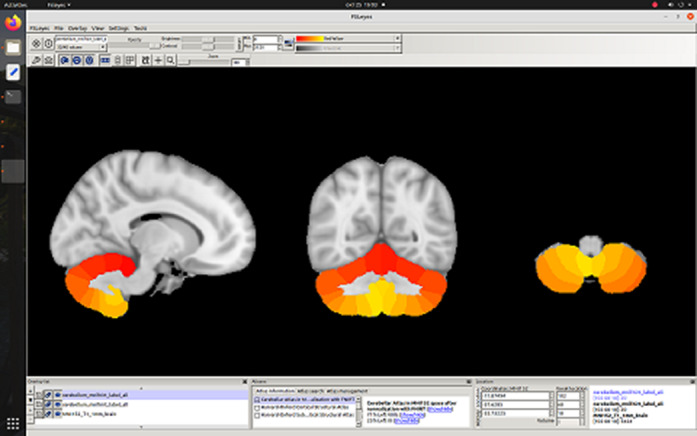

**Conclusions:**

To our knowledge, the absence of cerebellar myelination disorders in patients at an early-stage schizophrenia is reported for the first time, while the observed decrease in cerebrum myelination in schizophrenia is consistent with the previous findings. The difference in myelination between cerebellum and cerebrum may help to characterize the dynamics of the pathological process and provide additional information for understanding the biological mechanisms of the development of schizophrenia.

Grant RSF 20-15-00299 (partially).

**Disclosure of Interest:**

None Declared

